# The effects of CO_2_ and H_2_ on CO metabolism by pure and mixed microbial cultures

**DOI:** 10.1186/s13068-017-0910-1

**Published:** 2017-09-16

**Authors:** Sofia Esquivel-Elizondo, Anca G. Delgado, Bruce E. Rittmann, Rosa Krajmalnik-Brown

**Affiliations:** 1 Biodesign Swette Center for Environmental Biotechnology, Arizona State University, P.O. Box 875701, Tempe, AZ 85287-5701 USA; 2 School of Sustainable Engineering and the Built Environment, Arizona State University, Tempe, AZ USA

**Keywords:** Carbon monoxide, Syngas, CO-enriched mixed culture, *Acetobacterium*, Bioethanol, *Pleomorphomonas*, *Geosporobacter*

## Abstract

**Background:**

Syngas fermentation, the bioconversion of CO, CO_2_, and H_2_ to biofuels and chemicals, has undergone considerable optimization for industrial applications. Even more, full-scale plants for ethanol production from syngas fermentation by pure cultures are being built worldwide. The composition of syngas depends on the feedstock gasified and the gasification conditions. However, it remains unclear how different syngas mixtures affect the metabolism of carboxidotrophs, including the ethanol/acetate ratios. In addition, the potential application of mixed cultures in syngas fermentation and their advantages over pure cultures have not been deeply explored. In this work, the effects of CO_2_ and H_2_ on the CO metabolism by pure and mixed cultures were studied and compared. For this, a CO-enriched mixed culture and two isolated carboxidotrophs were grown with different combinations of syngas components (CO, CO:H_2_, CO:CO_2_, or CO:CO_2_:H_2_).

**Results:**

The CO metabolism of the mixed culture was somehow affected by the addition of CO_2_ and/or H_2_, but the pure cultures were more sensitive to changes in gas composition than the mixed culture. CO_2_ inhibited CO oxidation by the *Pleomorphomonas*-like isolate and decreased the ethanol/acetate ratio by the *Acetobacterium*-like isolate. H_2_ did not inhibit ethanol or H_2_ production by the *Acetobacterium* and *Pleomorphomonas* isolates, respectively, but decreased their CO consumption rates. As part of the mixed culture, these isolates, together with other microorganisms, consumed H_2_ and CO_2_ (along with CO) for all conditions tested and at similar CO consumption rates (2.6 ± 0.6 mmol CO L^−1^ day^−1^), while maintaining overall function (acetate production). Providing a continuous supply of CO by membrane diffusion caused the mixed culture to switch from acetate to ethanol production, presumably due to the increased supply of electron donor. In parallel with this change in metabolic function, the structure of the microbial community became dominated by *Geosporobacter* phylotypes, instead of *Acetobacterium* and *Pleomorphomonas* phylotypes.

**Conclusions:**

These results provide evidence for the potential of mixed-culture syngas fermentation, since the CO-enriched mixed culture showed high functional redundancy, was resilient to changes in syngas composition, and was capable of producing acetate or ethanol as main products of CO metabolism.

**Electronic supplementary material:**

The online version of this article (doi:10.1186/s13068-017-0910-1) contains supplementary material, which is available to authorized users.

## Background

Microbial anaerobic conversion of CO or synthesis gas (syngas), a gas mixture mainly composed of CO, H_2_, and CO_2_, leads to the production of important industrial products, such as acetic and butyric acid, and biofuels, such as ethanol, butanol, H_2_, and methane [1–4]. Pure cultures, mainly *Clostridium* spp. and other Clostridia, have been widely utilized in the study of CO and syngas conversion to ethanol and acetate [5–11]. Stoichiometric reactions for microbial conversion of CO and syngas to acetic acid and ethanol are summarized in Table 1; Eqs. 1–7.

**Table 1:** Stoichiometric reactions for microbial conversion of CO and syngas to acetic acid and ethanol

	$\Delta G^{^\circ \prime }$ (kJ/e^−^ eq.)^a^	
CO + H_2_O → H_2_ + CO_2_	− 10	Equation 1
4CO + 2H_2_O → CH_3_COOH + 2CO_2_	− 19.6	Equation 2
6CO + 3H_2_O → CH_3_CH_2_OH + 4CO_2_	− 18.6	Equation 3
2CO + 2H_2_ → CH_3_COOH	− 14.7	Equation 4
2CO + 4H_2_ → CH_3_CH_2_OH + H_2_O	− 18.0	Equation 5
4H_2_ + 2CO_2_ → CH_3_COOH + 2H_2_O	− 9.7	Equation 6
6H_2_ + 2CO_2_ → CH_3_CH_2_OH + 3H_2_O	− 8.7	Equation 7

e^−^ eq., electron equivalents

^a^Calculated from free energies of formation at 25 **°**C, pH = 7.0, and electron equivalency of moles of electron donor (i.e., CO and/or H_2_) reported in (Rittmann and McCarty [46])

Efforts to enhance CO and syngas conversion by carboxidotrophic microorganisms to preferred industrial products (e.g., ethanol or acetate) include optimization of nutrients [12, 13], optimization of pH and temperature [14, 15], optimization of bioreactor configuration [5, 9, 16, 17], and metabolic engineering for increased production [8, 18]. However, syngas composition varies depending on the feedstock gasified and the gasification conditions [19, 20]. Hence, different syngas compositions, including CO:CO_2_, CO:H_2_, and CO:CO_2_:H_2_ at different ratios with or without a source of organic carbon (e.g., yeast extract), varies in most studies [6, 15, 21–24], making it difficult to understand how CO_2_, H_2_, and different syngas mixtures affect CO oxidation and product distribution (e.g., ethanol/acetate ratios).

The available literature on this subject shows that growth of carboxidotrophic acetogens and the ethanol/acetate ratio are influenced by CO_2_ [7, 25–28]. In particular, several carboxidotrophs, including *Clostridium carboxidivorans*, do not grow with CO in the absence of CO_2_ in certain conditions [7]. Moreover, increasing the CO partial pressure (P_CO_) and the CO/CO_2_ ratio resulted in increased average cell concentration and increased ethanol/acetate ratio in *C. carboxidivorans* P7 [25]. Likewise, increased syngas pressure promoted ethanol production in *Clostridium ljungdahlii* [6]. Increased P_CO_, CO/CO_2_, or syngas pressure promoted ethanol production, possibly because more electrons as CO and/or H_2_ were available for reduction reactions. As seen in Eqs. 2–7 of Table 1, ethanol production requires more electrons as CO and H_2_ than acetate production.

The effect of H_2_ on CO fermentation has been less studied [28]. Although the conversion of CO and H_2_ to acetate or ethanol is thermodynamically feasible (Eqs. 4–5), only a few reports document pure cultures simultaneously consuming H_2_ and CO [29–31], mainly because CO inhibits hydrogenases [26, 32]. Accordingly, sustainable syngas conversion by pure cultures poses a challenge of keeping an optimal syngas composition in order to assure CO, H_2_, and CO_2_ consumption and the desired products ratio.

Mixed cultures are known to have functional redundancy and be more resilient to changes in the environment [33]. Moreover, industrial mixed-culture fermentation might be less costly than pure culture fermentation, as sterile conditions are not as stringent. Despite these potential advantages, little attention has been given to the use of mixed cultures for syngas fermentation [27, 34–36]. Similar to pure cultures, syngas components may have effects on the productivity of mixed cultures. Whether or not these effects are similar to the effects of syngas composition on pure cultures remains to be elucidated.

In this work, we studied and compared CO consumption rates and functionality of CO-consuming pure and mixed cultures during fermentation of CO and CO-rich mixtures commonly used in syngas studies (CO:CO_2_, CO:H_2_, and CO:CO_2_:H_2_). We grew a CO-enriched mixed culture and two carboxidotrophs isolated from it with the same CO-rich gas mixtures and similar conditions. We hypothesized that the addition of CO_2_ and/or H_2_ to CO fermentation would alter the metabolism of carboxidotrophs, regarding CO consumption rates and product formation, and that the pure cultures would be more susceptible to changes in gas composition than the mixed culture. In addition, we tested the capability of the CO-enriched mixed culture to switch from acetate to ethanol production by increasing the availability of electrons (as CO), which should promote reduction reactions. We carried out these experiments at neutral pH and in a batch reactor with continuous CO supply through diffusive membranes.

## Methods

### CO-enriched mixed culture and isolation of carboxidotrophs

A CO-enriched mixed culture was obtained by exposing anaerobic sludge to CO as the sole carbon and energy source. Details on the enrichment process were previously published [37]. Isolates namely SVCO-15 (GenBak KY992591) and SVCO-16 (GenBank KY992590) were obtained from the CO-enriched culture [37].

### Growth with CO and different CO-rich gas mixtures by pure and mixed cultures

1 mL of a CO-consuming culture (mixed or pure isolate) in exponential growth was used to inoculate glass serum bottles (160 mL) with 50 mL reduced anaerobic medium and different substrates: CO, CO:CO_2_, CO:H_2_, CO:CO_2_:H_2_, or CO:yeast extract (YE). Before inoculation, serum bottles were autoclaved for 1 h at 121 °C. Medium was buffered with sodium phosphate (Na_2_HPO_4_) and potassium phosphate (KH_2_PO_4_) in cultures growing with CO, CO:H_2_, and CO:YE, and it was buffered with sodium bicarbonate (NaHCO_3_) in cultures growing with CO:CO_2_ and CO:CO_2_:H_2_. The initial pH in this set of experiments with different gas compositions was 7.3 ± 0.4. The compositions of phosphate- and bicarbonate-buffered media were similar to those described in [38, 39], except that FeCl_2_ and Na_2_SO_4_ were not added, and the trace mineral solution was ATCC Trace Mineral Supplement (Catalog No. MD-TMS). Fermentation of CO also was studied in medium not buffered to allow the pH to decrease. The composition of the non-buffered medium (pH = 7.5 ± 0.1) was similar in composition to the phosphate-buffered medium, except that phosphate salts were not added. All media were reduced with l-cysteine (0.4 mM) and Na_2_S (0.2 mM) and supplemented with Wolfe’s vitamin solution (ATCC, Vitamin Supplement, catalog no. MD-VS), 0.01% (v v^−1^). Resazurin was used as an O_2_ indicator at a concentration of 5 × 10^−5^% (w v^−1^).

Serum bottles were incubated at 30 °C and shaken horizontally at 125 rpm to increase CO dissolution. Ultra high pure CO (99.9% purity) and H_2_, N_2_:CO_2_ (80:20), and pre-mixed CO:CO_2_:H_2_ (40:30:30) (Praxair, Danbury, CT) were used to feed the cultures. Preparation of gaseous substrates and final ratios of each mixture were as described by Esquivel-Elizondo et al. [37]. The initial total pressure of the serum bottles was 147 kPa (1.45 atm). Every condition was tested in triplicate.

### Fermentation of CO in batch reactor with continuous CO supply

The setup of the batch reactor is illustrated in Additional file 1: Figure S1. 32 composite hollow-fiber membranes (Mitsubishi Rayon, Model MHF 200TL) (1.31 cm mL^−1^) were used to supply CO (at ≤30.4 kPa) by diffusion directly to the liquid media. The CO delivery rate was 12 ± 0.5 μmol h^−1^, estimated from the amounts of measured end-products. The total volume of the batch reactors was 230 mL, and the liquid was 50 mL of reduced phosphate-buffered medium. Prior to inoculation with 2 mL of the CO-enriched mixed culture derived from sludge, reactors were sparged with N_2_ (UHP, Praxair) for 10 min and sealed with butyl rubber and aluminum crimps. The batch reactors were placed on a stir-bar hot plate (VWR, #97042-642) operated at 32 ± 1 °C and 150 rpm. Initial partial pressure of CO in the headspace of the reactor was 0 kPa.

### Chemical analyses

Gases in the headspace and fatty acids and alcohols in the liquid phase were detected and quantified via gas and liquid chromatography, respectively, as previously described [37, 40]. Partial pressures were determined with Dalton’s Law of partial pressures: *P* = % gas in headspace (estimated through gas chromatography) × total pressure in the batch reactor (measured with an electronic gauge manometer). pH values were measured with a pH Benchtop Meter (Thermo Scientific, #9142BN). Organic matter was estimated by quantifying the chemical oxygen demand (COD) in 2 mL samples with COD vial tests (Hach, LR and HR TNTplus vial tests).

### DNA extraction and molecular biology analyses

Pellets made by centrifuging 1.5 mL sample at 13,200 rpm for 20 min using a micro-centrifuge 5415 D (Eppendorf, Hauppauge, NY) were used for DNA extraction. To increase total biomass and DNA yield, replicate pellets (stored at −80 °C) were thawed and combined for DNA extraction [37]. Prior to inoculation of serum bottles, the 16S rRNA gene of isolates SVCO-15 and SVCO-16 was directly amplified from genomic DNA for Sanger sequencing using the primer set 8F/1525R, and the PCR conditions are described in [37]. The PCR products were purified and directly sequenced and aligned as previously described [37].

The 16S rRNA gene copies present in DNA extracted from cultures were quantified via quantitative real-time PCR (qPCR) using the primer set 342F/1492R and the following amplification conditions: pre-denaturation at 95 **°**C for 10 min; 45 cycles of denaturation at 95 **°**C for 15 s, and combined annealing and extension at 60 **°**C for 1 min; an additional cycle of 95 **°**C for 15 s, and 60 **°**C for 15 s; and 20 min temperature increase from 60 **°**C to 95 **°**C [41]. Each reaction (20 μL) contained: 10 μL of SYBR green mix (Takara, California), 0.2 μL of each forward and reverse primer (10 µM), 8.6 μL water, and 1 μL of template DNA (diluted 100×). Every assay and a six-point standard curve were run in triplicate. The gene copy number per mL was calculated with the following equation:

$${\text{gene copies per mL}} = \frac{( {\text{gene copies}}/{\text{reaction}} ) (\upmu{\text{L DNA}}^{\wedge}) ({\text{dilution factor}})}{{({\upmu {\text{L DNA}}/{\text{reaction}}} ) ({\text{mL sample}}^{*} )}}$$

*Volume of culture (sample) used for DNA extraction. ^^^Volume of DNA extracted from the sample.

To determine the bacterial community structures for the different conditions, DNA was sequenced using the Illumina MiSeq platform at the Microbiome Analysis Laboratory (http://krajmalnik.environmentalbiotechnology.org/microbiome-lab.html, Arizona State University, Tempe), using Bacterial primers 515F and 806R, which amplify the V4 hyper-variable regions of the 16S rRNA gene [42], and paired-end, 150 bp reads (2 × 150 mode). Raw sequences were submitted to NCBI Sequence Read Archive and are available under the Project ID PRJNA386595. Forward and reverse sequences were first paired (>45 bp overlap) using PANDASeq [43]. Then, paired reads (average length 253 bp) were processed using the QIIME 1.9.0 pipeline [44] as described in [40]. The average number of high-quality reads per sample was 43,938 ± 10,513.

### Electron balances

Electron balances were performed in order to understand the distribution of electrons from the electron donor(s) (CO, and/or H_2_) to end-products (acetate, ethanol, H_2_) [45]. Additional electrons, provided as yeast extract, l-cysteine (used as reducing agent), and biomass (or organic matter), also were considered. These electron balances were quantified as COD using 8 g COD/e^−^ eq. For the electron balances, mmol of electron donors and end-products were converted to electron milliequivalents (e^−^ meq.) using electron equivalents per mole values [46]. The distribution of e^−^ meq. to end-products is reported as a percentage of the initial total e^−^ meq. provided as substrate.

## Results and discussion

### Addition of H_2_ and/or CO_2_ to CO fermentation altered the metabolism of carboxidotrophs

The conversions of CO and CO with H_2_ and/or CO_2_ by a CO-enriched mixed culture and two carboxidotrophs isolated from the mixed culture were studied and compared. Based on the 16S rRNA gene partial sequence, the two isolates tested are 99% phylogenetically similar to the acetogen *Acetobacterium wieringae* and to the N-fixating bacterium *Pleomorphomonas diazotrophica* [37]. Despite these bacteria having not previously been characterized as carboxidotrophs, sequences mostly similar to *A. wieringae* were identified in a CO fermenter inoculated with anaerobic digester fluid from a wastewater treatment plant [47]. Additionally, we isolated another strain of *A. wieringae* from sediments collected close to a lake in Arizona, USA, enriched with CO (GenBank KY992593).

**Fig. 1:**
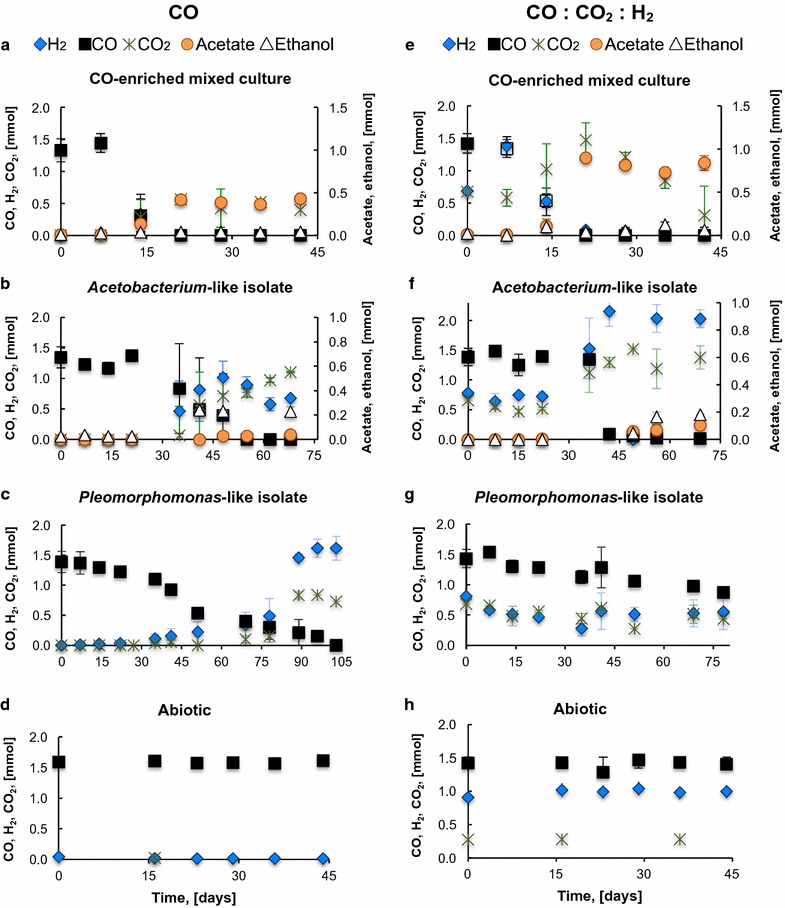
Fermentation of (**a**–**d**) CO, and (**e**–**h**) CO: CO_2_: H_2_ by the mixed and pure CO-consuming cultures. Panels **d** and **h** show that no CO, H_2_, or CO_2_ was abiotically consumed. The initial CO partial pressure was 30.4 kPa. The data are averages of triplicates; error bars indicate one standard deviation

**Fig. 2:**
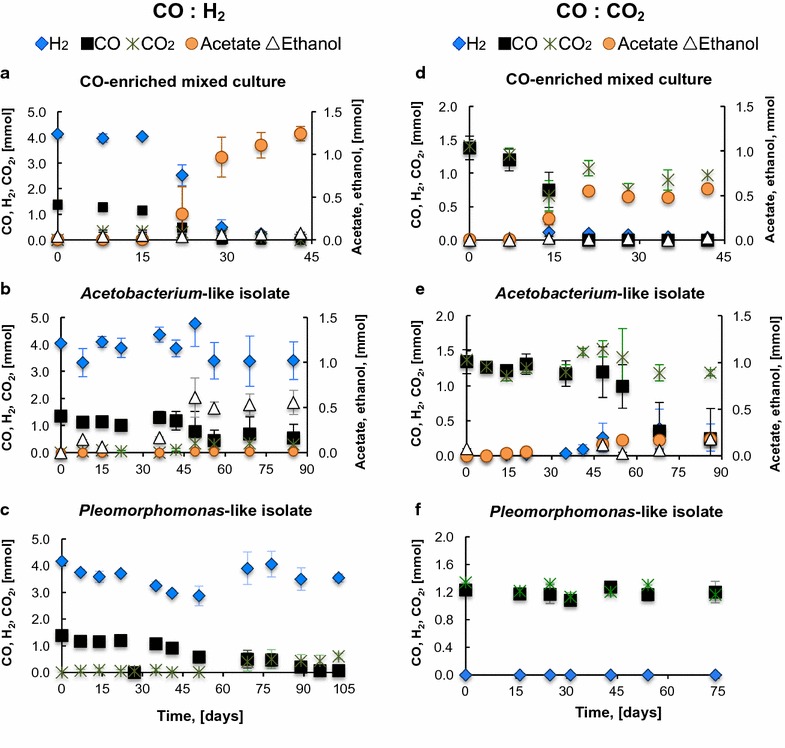
Fermentation of (**a**–**c**) CO: H_2_ and (**d**–**f**) CO: CO_2_ by the mixed and pure CO-consuming cultures. The initial partial pressure of CO was 30.4 kPa. The data are averages of triplicates; error bars indicate one standard deviation

Figures 1 and 2 present the conversion of CO-rich gas mixtures (i.e., CO, CO:CO_2_:H_2_, CO:H_2_, and CO:CO_2_,) by the CO-enriched mixed culture and the isolates. The mixed culture and the *Acetobacterium*-like isolate produced H_2_, CO_2_, acetate, and ethanol for all conditions tested. However, ethanol production by the *Acetobacterium* isolate was higher than by the CO-enriched mixed culture. While the isolate produced 0.56 and 0.18 mmol under growth with CO:H_2_ and CO:CO_2_, respectively (Fig. 2b, e), the CO enrichment produced 0.08 and 0.02 mmol (Fig. 2a, d). The *Pleomorphomonas*-like isolate produced H_2_ and CO_2_ from CO, according to the water-gas shift reaction (Eq. 1). No other products were detected via gas and liquid chromatography. As presented in Fig. 1d, h, abiotic conversion of CO, CO_2_, and H_2_ was negligible.

Opposite to the isolates, which did not completely consume H_2_, and previous studies with pure cultures, in which H_2_ was not consumed along with CO (Eqs. 4–5) [26, 28, 48, 49], the CO-enriched mixed culture metabolized H_2_ along with CO for acetate and ethanol production during growth with CO:CO_2_:H_2_ (Fig. 1e) and CO:H_2_ (Fig. 2a). Since CO dehydrogenase (CODH), the enzyme that catalyzes the reversible reduction of CO to CO_2_, possesses hydrogenase activity, the activity of hydrogenases in pure cultures of carboxidotrophs could be redundant [48]. However, H_2_ in syngas could have been consumed through Eqs. 6–7 (Table 1) by hydrogenotrophic microorganisms in the mixed culture. So far, the mesophilic proteobacterium *Rubrivivax gelatinosus* and the thermophilic *Methanothermobacter marburgensis* and *Thermoanaerobacter kivui* are reported to simultaneously utilize CO and H_2_. More experiments are needed to verify the simultaneous utilization of these substrates [29–31].

The presence of CO_2_ (0.7–1.4 mmol) and H_2_ (0.7–4.1 mmol) influenced the metabolism of the CO-consuming cultures. As summarized in Table 2, differences in CO consumption rates and ethanol/acetate ratios were observed when the gas components were varied. The pure cultures seemed more sensitive to the addition of H_2_ and/or CO_2_ to CO fermentation than the mixed culture. While maximum CO consumption rates (during exponential CO consumption) by the CO-enriched mixed culture were similar under all conditions (2.6 ± 0.6 mmol CO L^−1^day^−1^), those achieved by the isolates varied depending on the CO-rich gas mixture (0–5 mmol CO day^−1^ L^−1^). Moreover, contrary to the CO-enriched mixed culture, the final 16S rRNA gene copy number, quantified through qPCR, in pure cultures varied by more than one order of magnitude among the different CO-rich gas mixtures. Results from the qPCR analysis are presented and discussed in the supplemental material (Additional file 1: Figure S2).

**Table 2:** Electron equivalents in substrates and products, along with ethanol-to-acetate ratios, in the fermentation of CO and CO with H_2_ and/or CO_2_ by the CO-consuming cultures

CO-consuming culture	Gas mixture	Initial P_CO_, kPa	Initial P_CO2_, kPa	Initial P_H2_, kPa	Electrons in susbtrate^*^, e^−^ meq.	Initial C:e^−^ ratio, mmol/e^−^ meq.	H_2_, e^−^ meq.	Ethanol/acetate, e^−^ meq./e^−^ meq.	CO consumption rate^**^, mmol CO L^−1^ day^−1^
CO-enriched mixed culture	CO	0^a^	0	0	21.0^b^	–	0.1	0.12 - 0.3	3.4
					48.2^c^	–	0	–^d^	7.1
		31.4	0	0	4.1 ± 0.1	0.5	0.04	0.14 ± 0.03	3.5
	CO: H_2_	31.4	0	96.2	9.4 ± 0.3	0.2	0	0.03 ± 0.01	2.4
	CO: CO_2_	32.4	32.4	0	4.1 ± 0.0	0.7	0.2 ± 0.1	0.03 ± 0.03	2.5
	CO: CO_2_: H_2_	32.4	15.2	16.2	6.7	0.5	0	0.06 ± 0.03	2.1
*Acetobacterium*-like isolate	CO	30.4	0	0	4.1 ± 0.1	0.5	1.8 ± 0.3	14.2 ± 5.2	1.4
	CO:YE	30.4	0	0	6.0 ± 0.0	0.3	1.0 ± 0.01	400 ± 133.3	1.8
	CO:H_2_	30.4	0	92.2	9.4 ± 0.3	0.2	1.3 ± 0.1	11.2 ± 0.5	0.7
	CO:CO_2_	30.4	31.4	0	4.1 ± 0.0	0.7	0.5 ± 0.3	0.86 ± 0.01	1.4
	CO:CO_2_:H_2_	31.4	15.2	17.2	5.8	0.5	2.5 ± 0.3	2.5 ± 0.25	5.0
*Pleomorphomonas*-like isolate	CO	31.4	0	0	4.1 ± 0.1	0.5	3.2 ± 0.2	–	1.0
	CO: YE	29.4	0	0	4.7 ± 0.1	0.3	3.8 ± 0.3	–	1.3
	CO: H_2_	30.4	0	95.2	9.4 ± 0.3	0.2	7.1 ± 0.15	–	0.7
	CO: CO_2_	28.4	30.4	0	4.1 ± 0.1	0.7	0.0	–	0.0
	CO: CO_2_:H_2_	30.4	15.2	18.2	3.4 ± 0.04	0.5	1.0 ± 0.3	–	1.1

* Electrons added as CO and/or H_2_, and organic matter (i.e., fatty acids in inoculum)

** Maximum rates achieved during exponential CO consumption in serum bottles, and estimated rates in the batch reactor

^a^Initial P_CO_ in batch reactor with continuous CO supply is 0 kPa, since CO gradually diffuses through the membranes, and only CO not consumed by the biofilm formed on the membranes ends in the headspace of the reactor

^b^After 55 days of fermentation, at P_CO_ = 12.16 kPa

^c^After 61 days of fermentation, at P_CO_ = 4.05 kPa

^d^No acetate detected, and 69.5 ± 3.1 mM (40.6 e^−^ meq.) of ethanol produced

**Fig. 3:**
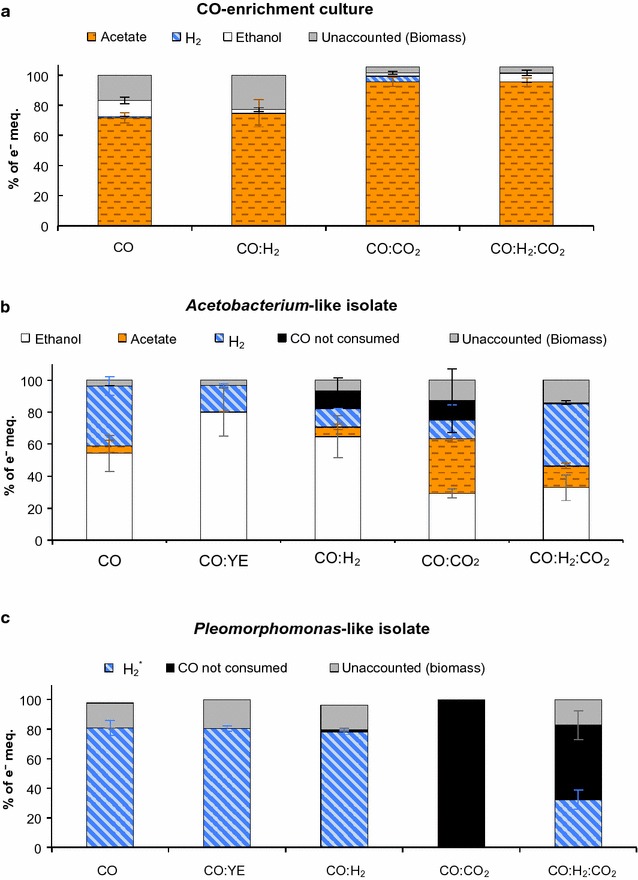
Electron distribution (%) from electron donors^a^ to acetate, ethanol, and/or H_2_ produced in fermentation of CO and mixtures of CO with CO_2_ and/or H_2_ by (**a**) the CO-enriched mixed culture, (**b**) the *Acetobacterium*-like isolate, and (**c**) the *Pleomorphomonas*-like isolate. The data are averages with standard deviation of triplicates. The electron balance in (**a**) corresponds to day 21, and in (**b**, **c**) to the last day of fermentation. ^a^CO, H_2_, and/or yeast extract (YE), and initial concentration of organic matter. ^*^Distribution of electrons to H_2_ includes H_2_ produced and not consumed. e^−^ meq.: electron milliequivalent

Production of acetate, ethanol, and/or H_2_ from CO-rich gases by the CO-consuming cultures is compared in the electron balances presented in Fig. 3. For this, end-products electron equivalents were normalized by the initial electron equivalents in CO and/or H_2_ and COD in the inoculum, as summarized in Table 2. Figure 3a shows that acetate was the dominant end-product in all fermentations by the CO-enriched mixed culture. More than 72% of the electrons in substrates were utilized for acetate production. Ethanol was produced for all conditions tested, although at smaller proportions: 11 ± 2.3% of the electrons in CO and 2–6% of the electrons in CO with CO_2_ and/or H_2_ were directed towards ethanol production. Initial pH was 7.3 ± 0.3, and it decreased to 6.8 ± 0.13 at the end of the experiments. In addition, the final ethanol/acetate ratio was similar for growth with CO_2_ and H_2_ as substrates (0.04 ± 0.015 e^−^ meq./e^−^ meq.), and it increased to 0.14 ± 0.03 e^−^ meq./e^−^ meq. for growth with CO as the sole substrate (Table 2). Similarly, estimated ethanol/acetate ratios by an acetogenic mixed culture were 0.37 e^−^ meq./e^−^ meq. at P_CO_ = ~76 kPa (no CO_2_ added) and decreased to 0.08 and 0.07 e^−^ meq./e^−^ meq. at P_CO_ = 25.3 kPa and P_CO_ and P_CO2_ of ~25.3 kPa, respectively [27].

Figure 3b shows that the electron distribution to products by the *Acetobacterium*-like isolate varied depending on the presence of H_2_ and/or CO_2_. The highest distribution of electrons to ethanol was achieved with CO and CO:H_2_: 54–64% of the electrons in CO:H_2_ or CO were utilized for ethanol production, whereas less than 33% of the electrons in CO:CO_2_:H_2_ and CO:CO_2_ went to ethanol. The rest of the electrons were distributed to biomass, acetate, and H_2_, also in different proportions. Similar to the CO-enriched mixed culture, the highest fraction of electrons channeled to acetate by the *Acetobacterium*-like isolate was achieved with the addition of CO_2_. Initial medium pH was 7.3 ± 0.3, and it decreased to 7.1 ± 0.0, 7.0 ± 0.1, 7.0 ± 0.1, 6.6 ± 0.2, and 6.8 ± 0.1 after fermentation of CO, CO:YE, CO:H_2_, CO:CO_2_, and CO:CO_2_:H_2_, respectively. The different final pH values are in accordance with the different ethanol/acetate ratios observed (Table 2); final lower pH corresponded to higher acetate concentrations. Accordingly, ethanol/acetate ratios obtained with growth with CO:CO_2_ and CO:CO_2_:H_2_ were at least 5 times lower (0.86 ± 0.01 and 2.5 ± 0.25 e^−^ meq./e^−^ meq., respectively) than those achieved with CO and CO:H_2_ (14.2 ± 5.2 and 11.2 ± 0.5 e^−^ meq./e^−^ meq., respectively) (Table 2). These results agree with the results of Heiskanen et al. [28] and Nam et al. [27], who report an increase in the acetate production rate and yield with the addition of CO_2_ to CO fermentation. Ethanol/acetate ratios by the *Acetobacterium*-like isolate (at total pressures of 147 kPa) growing in syngas (2.5 ± 0.25 e^−^ meq./e^−^ meq.) are higher than those reported for *Clostridium ljungdahlii* (~0.1–1 e^−^ meq./e^−^ meq.) growing with syngas (55% CO, 10% CO_2_, 20% H_2_, 15% Ar) at 101.3–182.4 kPa [6], but lower to that (~8.5 e^−^ meq./e^−^ meq.) achieved by *C. ljungdahlii* in a 4-L bioreactor optimized for solventogenesis [50]. In our study, the addition of H_2_ also promoted acetate production by the CO-enriched mixed culture, but not by the *Acetobacterium* isolate. A possible explanation is that, while the isolate did not consume most of the H_2_ provided as substrate (Fig. 2b), the CO-enriched mixed culture consumed H_2_ along with CO (Fig. 2a), for increased acetate production.

The addition of CO_2_ and/or H_2_ to CO fermentation also influenced the metabolism of the *Pleomorphomonas*-like isolate. CO conversion was highly inhibited by the presence of CO_2_ in growth with CO:CO_2_ (P_CO2_ = 30.4 kPa) and CO:CO_2_:H_2_ (P_CO2_ = 15.2 kPa). More than 70% of the added CO was not consumed after >75 days of fermentation (Figs. 1g, 2f). Exogenous H_2_ (P_H2_ = 95.2 kPa) did not inhibit CO consumption, and similar to the CO-enriched mixed culture, it decreased the CO consumption rates, from 1.0 to 0.7 mmol CO L^−1^ day^−1^, compared to autotrophic growth with CO as the sole substrate. Correspondingly, the distribution of electrons from CO to H_2_ was similar (78–81%) in growth with CO and CO:H_2_, but different than in growth with CO:CO_2_ and CO:CO_2_:H_2_ (Fig. 3c).

Many carboxidotrophs have been observed to require or grow better with yeast extract or similar complex nutrients [13, 51]. The addition of yeast extract (0.05% w v^−1^) significantly (*P* < 0.01) increased ethanol production by the *Acetobacterium*-like isolate, compared to its autotrophic growth with CO as the sole substrate (from 6.6 ± 1.0 to 13.5 ± 3.0 mM). This increase in ethanol production can be seen in Fig. 3b: the distribution of electrons to ethanol increased from 54 ± 11% in growth with CO, to 80 ± 16% in growth with CO and yeast extract, although similar maximum CO consumption rates were achieved: 1.6 ± 0.2 mmol CO L^−1^ day^−1^ (Table 2). Acetate production by the *Acetobacterium*-like isolate grown with CO:YE was not detected (Additional file 1: Figure S3A). Similarly, CO consumption by the *Pleomorphomonas*-like isolate was stimulated by the addition of yeast extract (0.05% w/v); total CO was consumed in less than half of the time when yeast extract was added (Fig. 1c; Additional file 1: Figure S3B). However, more electrons were provided as substrate with the addition of yeast extract; therefore, more biomass was produced. The higher concentration of biomass was possibly responsible for the shorter lag phase for CO consumption observed in growth with CO:YE compared to CO. However, once microbes were in exponential growth phase, maximum CO conversion rates (1.2 ± 0.2 mmol CO L^−1^ day^−1^) were similar (Table 2). As seen in Fig. 3c, the electron distribution to H_2_ by the *Pleomorphomonas*-like isolate grown with CO:YE was similar to that observed when grown with CO and CO:H_2_.

The metabolisms of the *Acetobacterium*- and *Pleomorphomonas*-like isolates were affected by changes in syngas composition. CO_2_ (at P_CO2_ = 30.4 kPa) inhibited CO oxidation by the *Pleomorphomonas* isolate and ethanol production by the *Acetobacterium* isolate. H_2_ (at P_H2_ = 91.2 kPa), on the other hand, did not inhibit ethanol or H_2_ production by the *Acetobacterium* and *Pleomorphomonas* isolates, respectively, but decreased their CO consumption rates. These results indicate that the metabolism of these isolates, and possibly of other microorganisms, depends on the syngas composition. Therefore, for industrial applications of non-engineered microorganisms, such as the studied isolates, the feedstock and gasification conditions should be constant in order to fix the syngas composition, and to maintain concentration of the desired end-product(s).

The *Acetobacterium* and *Pleomorphomonas* isolates may have been the main carboxidotrophs in the CO-enriched mixed culture that generated H_2_ and CO_2_, and acetate and ethanol, respectively, from CO (Figs. 1, 2, 3; Table 2; Additional file 1: Table S1). As part of the mixed culture, these isolates, together with other microorganisms summarized in Additional file 1: Figure S4, consumed CO along with CO_2_ and H_2_ at CO consumption rates of 2.6 ± 0.6 mmol CO L^−1^ day^−1^. These rates are similar to the ones achieved by thermophilic CO and syngas enrichment cultures (~1.3 – 2.8 mmol CO L^−1^ day^−1^ in [34]) and a mesophilic mixed culture (4 mmol CO L^−1^ day^−1^ in [27]), but lower than the rates achieved by sludge from a wastewater treatment plant treating paper mill (>10 mmol CO L^−1^ day^−1^ in [52]). Ethanol/acetate ratios achieved by the mesophilic CO-enriched mixed culture varied slightly for different combinations of syngas components. Regardless of these ratios and different structures of the microbial communities (Additional file 1: Figure S4), acetate always was the main end-product from CO, CO:CO_2_, CO:H_2_, and CO:CO_2_:H_2_ (Fig. 3a). Functional redundancy of mixed cultures is important for the sustainable production of acetate and other products from syngas, where the composition of syngas and, therefore, P_CO_, P_CO2_, and P_H2_, constantly vary depending on the type of feedstock gasified (e.g., recalcitrant biomass waste) and the gasification conditions [19, 20].

### Ethanol production by the CO-enriched mixed culture increased with continuous CO supply at pH ~7

Since the CO-enriched mixed culture showed advantages for industrial applications over pure cultures (i.e., resiliency to changes in syngas composition, complete syngas consumption at relatively fast CO consumption rates), we further investigated its capacity to produce ethanol over acetate. First, in order to test if a drop in pH would lead to a switch in metabolism from acetate production to ethanol production, as previously reported [15, 25, 35, 53], CO fermentation was carried out in serum bottles with non-buffered medium. The results, presented in Additional file 1: Figure S5, showed that pH decreased from 7.5 ± 0.1 to 6.8 ± 0.1 and that phosphate was necessary for fast CO conversion by the CO-enriched mixed culture, since CO consumption rates in non-buffered medium were slower (1.1 mmol CO day^−1^ L^−1^) than rates achieved in phosphate-buffered medium (2.1–3.5 mmol CO day^−1^ L^−1^). This agrees with the fact that the CO enrichment culture was enriched with CO in phosphate buffer media [37]. Results on ethanol production, pH values over time, and microbial phylotypes identified in these experiments are presented in the supplemental material.

**Fig. 4:**
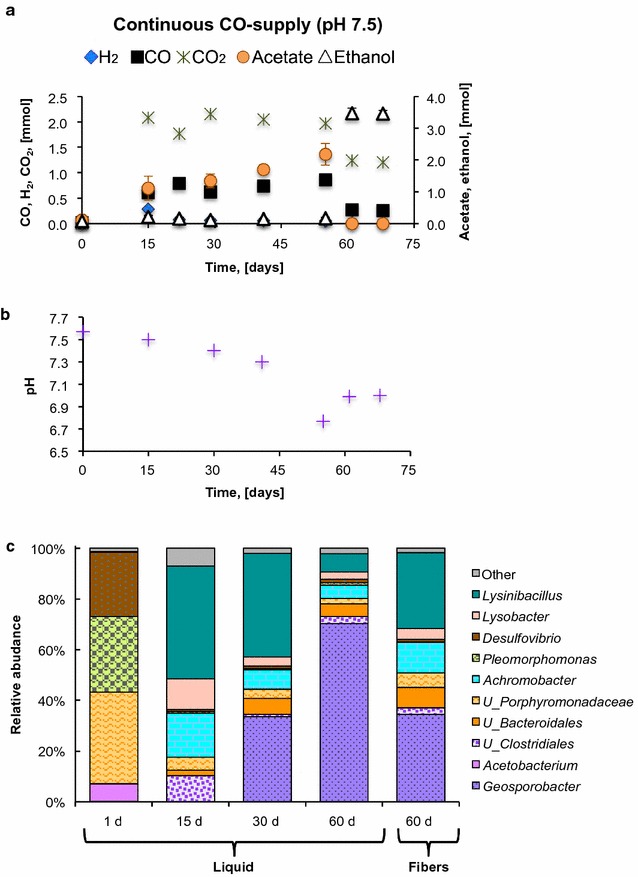
Fermentation of CO in a batch membrane reactor with continuous CO supply in phosphate-buffered medium. **a** H_2_, CO_2_, and CO measured in the headspace, and acetate and ethanol concentrations in the liquid phase. **b** pH values over time. **c** Relative abundance of main phylotypes detected during fermentation. The data in **a**, **b** are averages of at least two measurements

In order to enhance ethanol production at faster CO consumption rates, fermentation of CO in phosphate-buffered medium (pH 7.5 ± 0.1) was carried out in a batch reactor with continuous CO supply (Additional file 1: Figure S1). The hypothesis tested was that increased availability of electrons (as CO) would increase the reduction potential of the medium, promoting reduction reactions, including ethanol production, to maintain the redox balance. As shown in Fig. 4a and Table 2, acetate and ethanol were produced along fermentation with ethanol/acetate ratios between 0.12 and 0.3 e^−^ meq./e^−^ meq. However, on day 61, a switch from acetate to ethanol production was observed at a pH of 6.9 ± 0.1 (Fig. 4b). Ethanol concentrations increased from 3.4 mM (0.17 mmol) to 69.5 ± 3.1 mM (3.48 ± 0.16 mmol), whereas acetate was no longer detected. This high degree of ethanol production was maintained for 1 more week, until operation of the batch reactor was stopped. Continuous delivery of CO in a batch system increased the availability of electrons for reduction reactions, which promoted the reduction of the C=O group in acetate to a CH_2_ group to form ethanol. In this regard, increased ethanol production has been observed at higher CO and syngas partial pressures or reducing agent concentrations [6, 10, 25, 53].

As shown in Fig. 4c, the microbial community in the batch reactor with continuous CO supply was distinct from the microbial communities identified in serum bottles with CO and CO-rich mixtures (Additional file 1: Figure S4, S5C), despite being inoculated with the same mixed culture. Phylotypes associated with the acetate producer *Geosporobacter* (order *Clostridiales*) [54] were <0.1% abundant in serum bottles (with buffered and non-buffered media), but dominated the identified microbial community in the batch reactor, both in the liquid phase and in the fibers, after 30 days of fermentation. When the switch from acetate to ethanol production was observed, the relative abundance of *Geosporobacter* phylotypes, in the liquid phase, increased from 34 to 70%. *Porphyromonadaceae* (order *Bacteroidales*) were also detected at high abundance during fermentation with continuous CO supply (2–36%). Phylotypes associated with these acetate producers were also detected in the fermentation of CO and CO-rich gas mixtures in serum bottles, at considerable abundance (up to 4.4%, Additional file 1: Figures S4, S5C). Other phylotypes related to bacteria that ferment acetate became highly abundant in fermentation with continuous CO supply, including the aerobes *Achromobacter* and *Lysinibacillus*. These microbes might have been enriched with the small amounts of O_2_ introduced after sampling, or they might have facultative metabolism. Despite the abundance of these “contaminating” microorganisms, ethanol concentrations and CO consumption rates were high, underscoring the advantages of using mixed microbial communities for robust industrial applications. In summary, increasing the number of electrons (as CO) directly delivered to microorganisms increased ethanol production at neutral pH, and promoted a different microbial community structure, compared to CO fermentation in serum bottles.

## Conclusions

Addition of H_2_ and CO_2_ during CO fermentation affected the metabolism of the carboxidotrophs. Pure cultures obtained from a CO-enriched mixed culture were more sensitive to changes in syngas components than the CO-enriched mixed culture. The mixed culture showed several advantages over the pure cultures as it adjusted to changes in syngas components and consumed CO along with H_2_ and CO_2_. While acetate was the main end-product in serum bottles with a single addition of CO or syngas (P_CO_ = 31.4 kPa), the metabolism of the mixed culture switched to ethanol production, at neutral pH, after increasing the reduction potential of the medium through continuous CO supply in a batch reactor. These results provide useful insights towards the sustainable production of acetate, ethanol, and other products from syngas, particularly when the composition of syngas varies depending on the type of feedstock gasified and the gasification conditions.

## Additional file

**Additional file 1.** Pictures of the batch membrane reactor and additional results: qPCR analysis, growth with CO and yeast extract, community structure of the mixed culture, fermentation of CO in non-buffered medium, and carbon balance.

## Abbreviations

CO: carbon monoxide
CO_2_: carbon dioxide
H_2_: hydrogen
P_CO_: CO partial pressure
P_CO2_: CO_2_ partial pressure
P_H2_: H_2_ partial pressure
COD: chemical oxygen demand
YE: yeast extract
qPCR: quantitative real-time polymerase chain reaction
