# Supplementary material for: The effects of CO2 and H2 on CO metabolism by pure and mixed microbial cultures
Source: Biotechnol Biofuels. 2017 Sep 16;10:220. doi: 10.1186/s13068-017-0910-1 (PMC5603099; doi:10.1186/s13068-017-0910-1)
Supplement: Supplementary file 1 — Additional file 1. Pictures of the batch membrane reactor and additional results: qPCR analysis, growth with CO and yeast extract, community structure of the mixed culture, fermentation of CO in non-buffered medium, and carbon balance. [file 13068_2017_910_MOESM1_ESM.pdf]

The effects of CO_2 and H_2 on CO-metabolism by pure and mixed microbial cultures

Sofia Esquivel-Elizondo[1,2], Anca G. Delgado[1,2], Bruce E. Rittmann[1,2], and Rosa Krajmalnik-Brown[1,2#]

[1] *Biodesign Swette Center for Environmental Biotechnology, Arizona State University, P.O. Box 875701, Tempe, AZ 85287- 5701, USA*

[2]*School of Sustainable Engineering and the Built Environment, Arizona State University, Tempe, AZ, USA*

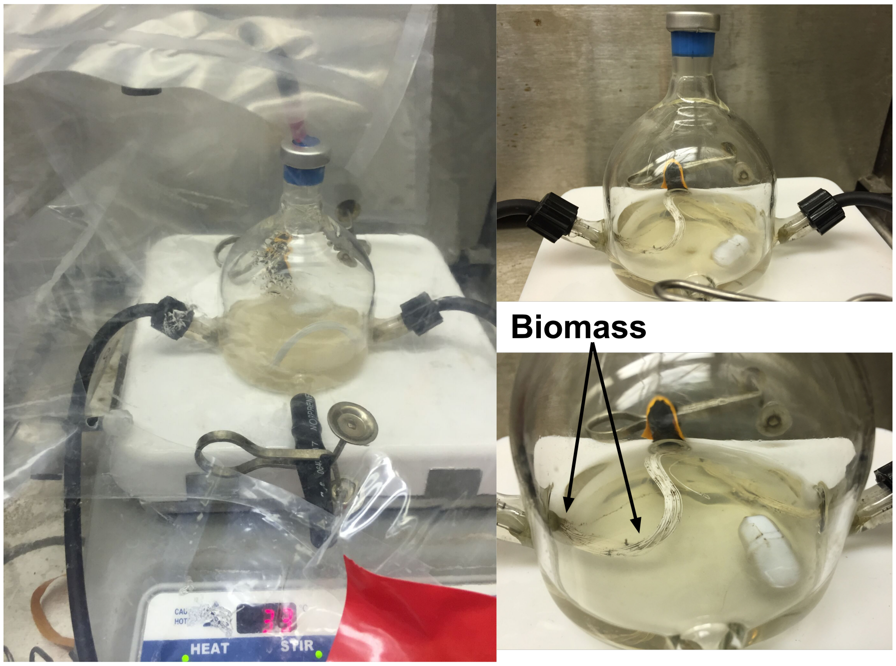

Figure S1. Batch reactor with continuous CO-supply to liquid medium through diffusive membranes.

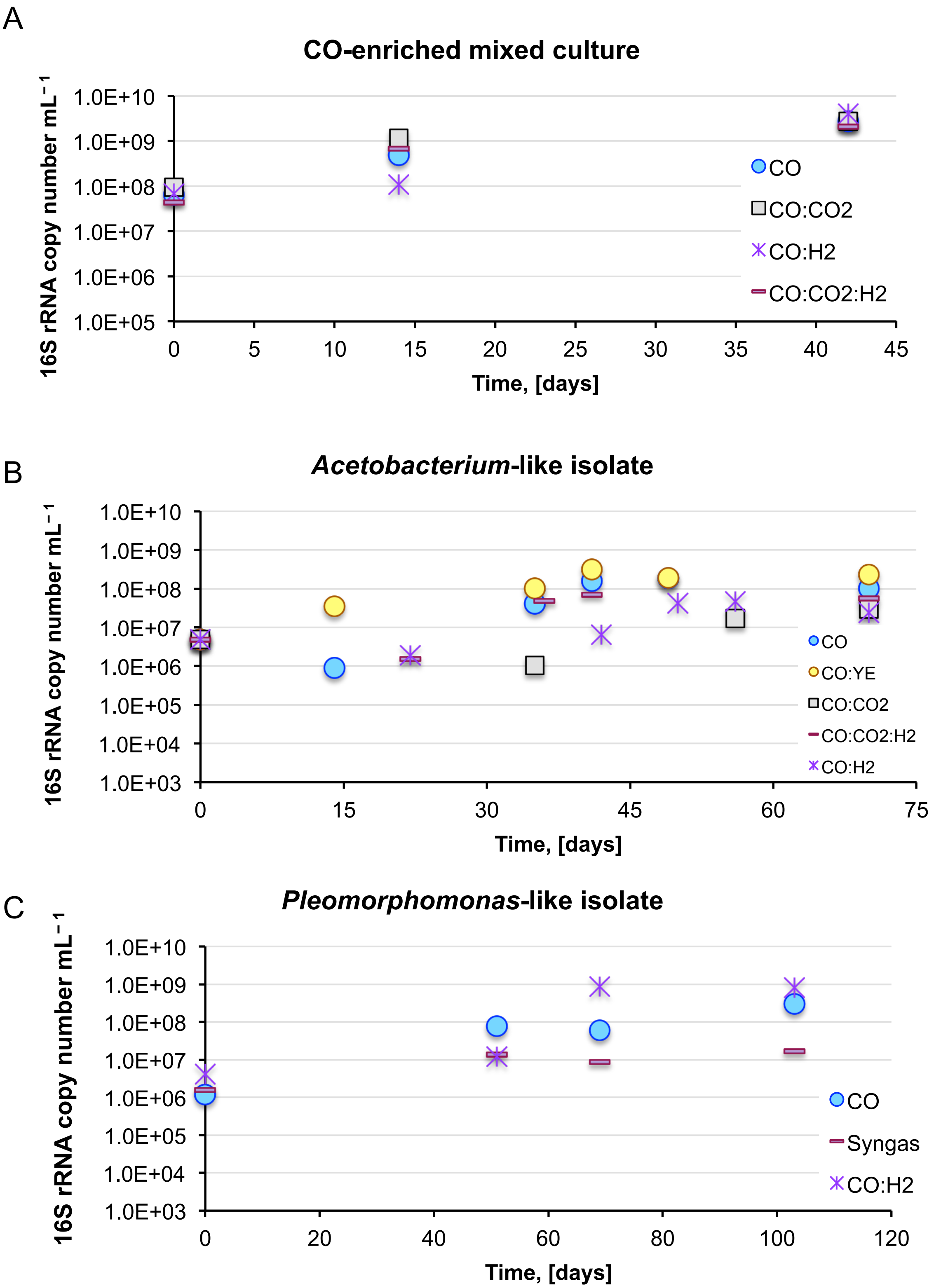

Figure S2. Copies of the 16S rRNA gene in the CO-consuming cultures: (A) CO-enrichment culture, (B) *Acetobacterium*-like isolate, and (C) *Pleomorphomonas*-like isolate growing with CO, and mixtures of CO with H_2 and/or CO_2. Values are averages of triplicate qPCR reactions on pooled DNA.

The metabolism of the CO-enriched mixed culture was not inhibited by the addition H_2 or CO_2 (Figures 1-A,E and 2-A,D). Accordingly, as seen in Figure S2-A, the final copy number of the 16S rRNA gene in the mixed culture, quantified through qPCR, was similar under all conditions tested (5.3 ± 1.8 × 10 ^ 8 copies mL^{-1}). This indicates that the differences in initial amount of electrons (~ 4.1 – 9.4 me^{-} eq.) in growth with CO or CO with CO_2 and/or H_2 were not enough to observe significant differences in the 16S rRNA gene copy number. However, as seen in Figure 2-B,C, the final 16S rRNA gene copy number in the pure cultures depended on the CO-rich mixture, and substrate consumption. After fermentation of CO (with and without yeast extract) by the *Acetobacterium*-like isolate, the final gene copy number increased 2 orders of magnitude (from 4.8 ± 1.1 × 10^6 to 1.7 × 10^8 ± 9.4 × 10^7 copies mL^{-1}), whereas in growth with CO:H_2, CO:CO_2, and CO:CO_2:H_2, copies of the 16S rRNA gene only increased 1 order of magnitude (4.1 ± 2.5 × 10^7 copies mL^{-1}) (Figure S2B). This smaller increase in copies of the gene is in accordance with the incomplete consumption of CO and/or H_2 observed in those autotrophies (Figures 1-B,F and 2-B,E). Similarly, copies of the 16S rRNA gene of the *Pleomorphomonas*-like isolate (Figure 2S-C) increased 2 orders of magnitude after fermentation of CO and CO:H_2 (from 2.7 ± 2 × 10^6 to 5.5 ± 3.6 × 10^8), where all CO was consumed, and less than one order of magnitude in fermentation of CO:CO_2:H_2 (8.8 × 10^6), where 70 % of the CO was not consumed.

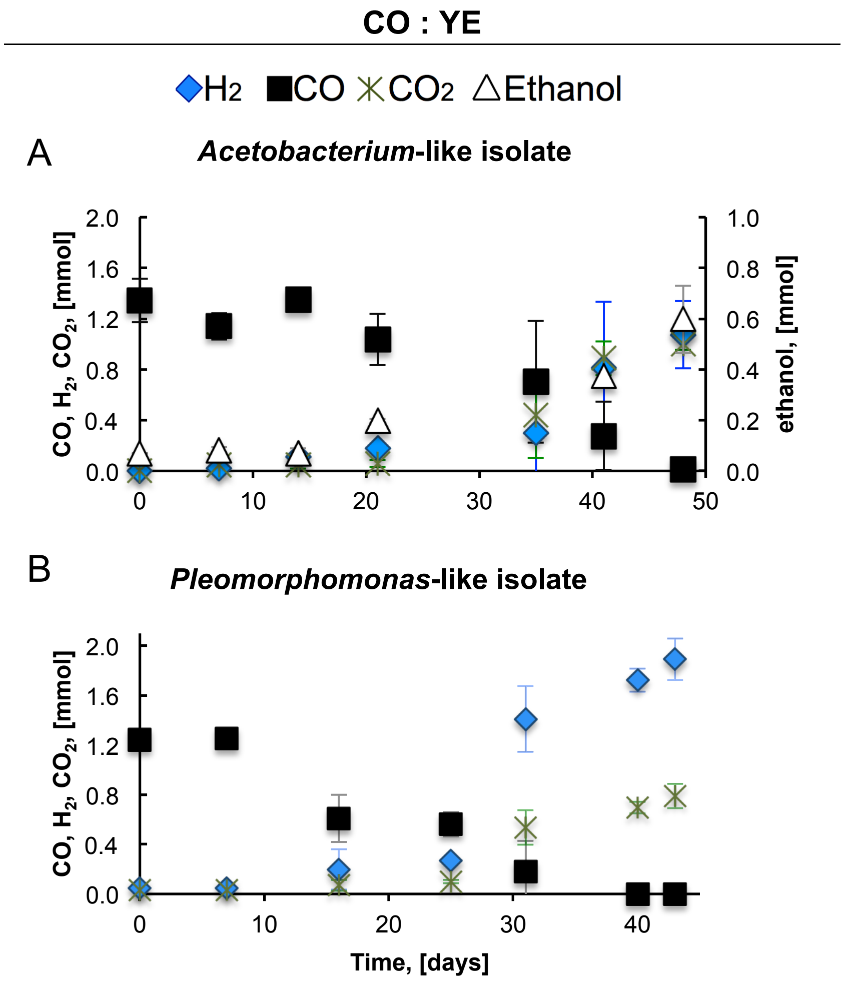

Figure S3. Fermentation of CO with yeast extract (0.05 % w/v) by A) the *Acetobacterium*-like isolate and B) the *Pleomorphomonas*-like isolate. The initial CO partial pressure was 30.4 kPa (0.3 atm). The data are averages of triplicates; error bars indicate one standard deviation. This is a modified figure from Esquivel-Elizondo et al., 2017.

Sofia Esquivel-Elizondo, Anca G. Delgado, Rosa Krajmalnik-Brown; Evolution of microbial communities growing with carbon monoxide, hydrogen, and carbon dioxide, *FEMS Microbiology Ecology*, Volume 93, Issue 6, 1 June 2017, fix076, https://doi.org/10.1093/femsec/fix076

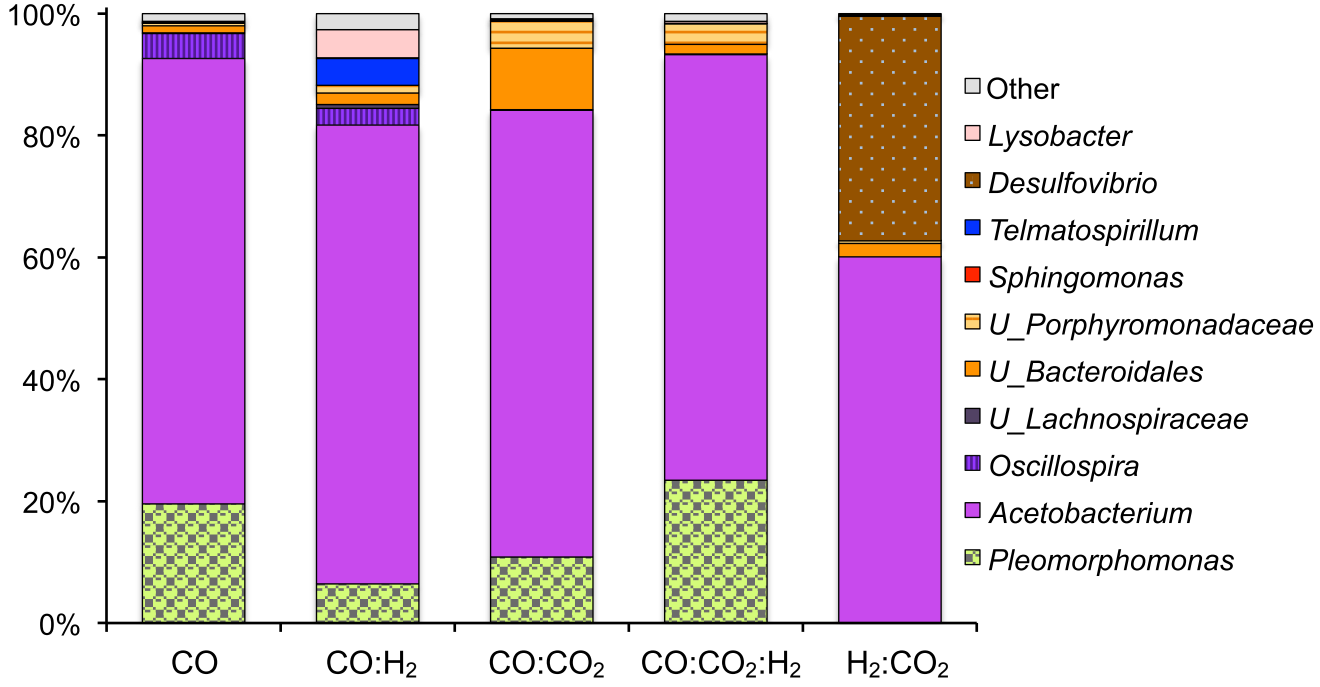

Figure S4. Relative abundance of main phylotypes detected at exponential CO-consumption phase during fermentation of CO, mixtures of CO with CO_2 and/or H_2, and H_2:CO_2 (control no CO) by the CO-enrched mixed culture. Initial CO partial pressure was 0.3 atm. The data are an average of triplicates.

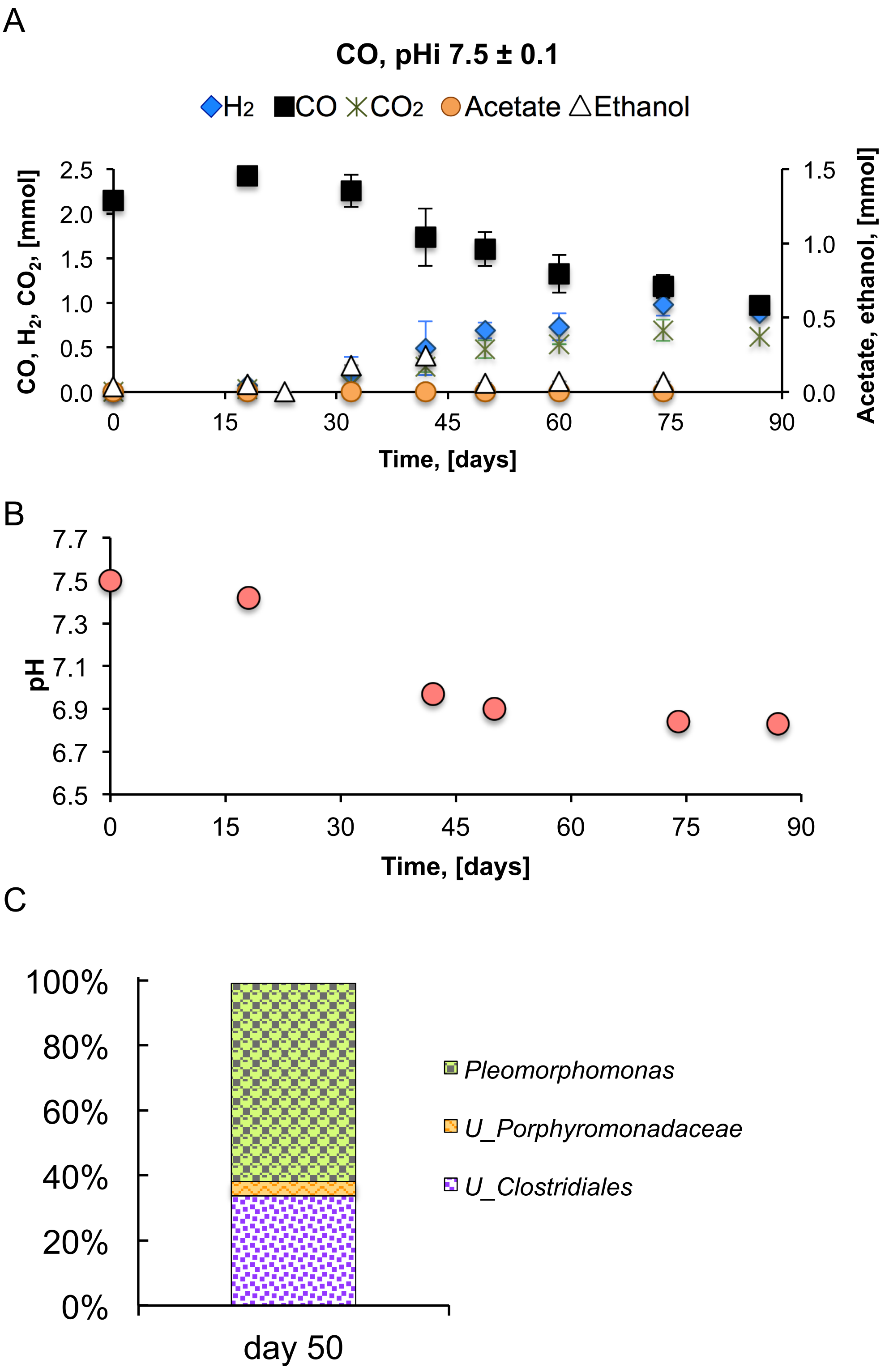

Figure S5. Fermentation of CO by the CO-enriched mixed culture in medium not buffered at initial pH (pHi) = 7.5 ± 0.1. A) Products from CO-metabolism. B) pH values over time. C) Main phylotypes detected on day 50 of fermentation.
"U_" stands for unidentified microorganism within the taxonomic classification.

Non-buffered medium was similar in composition to phosphate-buffered medium used in acetate production from different syngas mixtures, excepting the presence of phosphate salts. The non-buffered system allowed the pH to drop from 7.5 ± 0.1 to 6.8 ± 0.1. As seen in Figure S5-A, ethanol was detected during fermentation in non-buffered medium at concentrations of up to 5.4 ± 0.2 mM (~0.24 mmol), while acetate was not detected. Based on 16S rRNA community analysis, the main phylotypes detected after 50 days of fermentation, when 1.3 ± 0.05 mmol of CO were consumed, were those related to the carboxidotrophic hydrogenogen *Pleomorphomonas* (61% of the identified phylotypes), followed by unidentified bacteria associated with fermenters within the bacterial orders Clostridiales (34%) and Bacteroidales (4.4%) (Figure S5-B). *Acetobacterium*, *Oscillospira*, and other main phylotypes detected in phosphate-buffered medium (Figure S4) were not abundant. The absence of phylotypes related to acetate-producing bacteria coincides with the lack of acetate production observed. Although ethanol was produced, low concentrations were attained compared to other studies (1–4). This low ethanol production could be due to the slow CO-utilization rates and incomplete CO fermentation observed in medium not buffered: 1.1 mmol CO d^{-1} L^{-1}, compared to 2.1 – 3.5 mmol CO d^{-1} L^{-1} achieved with phosphate-buffered medium.

References

1. **Ganigue R**, **Ramio-Pujol S**, **Sanchez P**, **Bañeras L**, **Colprim J**. 2015. Conversion of sewage sludge to commodity chemicals via syngas fermentation. Water Sci Technol **72**:415–420.
2. **Mohammadi M**, **Younesi H**, **Najafpour G**, **Mohamed AR**. 2012. Sustainable ethanol fermentation from synthesis gas by Clostridium ljungdahlii in a continuous stirred tank bioreactor. J Chem Technol Biotechnol **87**:837–843.
3. **Abubackar HN**, **Veiga MC**, **Kennes C**. 2012. Biological conversion of carbon monoxide to ethanol: Effect of pH, gas pressure, reducing agent and yeast extract. Bioresour Technol **114**:518–522.
4. **Hurst KM**, **Lewis RS**. 2010. Carbon monoxide partial pressure effects on the metabolic process of syngas fermentation. Biochem Eng J **48**:159–165.

Table S1. Carbon balance.

CO-consuming culture	Gas mixture	C (substrates[a]), mmol	C (products[b]), mmol	% of C recovered
CO-enriched mixed culture	CO	1.4	1.3	94.4
	$CO:H_2$	1.4	1.9	142.8
	$CO:CO_2$	2.8	2.1	73.5
	$CO:CO_2:H_2$	2.1	2.2	105.2
Acetobacterium-like isolate	CO	1.4	1.6	113.8
	$CO:H_2$	1.4	1.4	105.9
	$CO:CO_2$	2.7	2.2	79.4
	$CO:CO_2:H_2$	2.2	1.9	90.6
Pleomorphomonas-like isolate	CO	1.4	0.7	52.9
	$CO:H_2$	1.4	0.7	49.1
	$CO:CO_2$	2.6	2.4	91.8
	$CO:CO_2:H_2$	2.1	1.3	61.6

[a] Carbon in gas mixture

[b] Carbon in products detected through gas and liquid chromatography

Note: Only carbon in gas substrates and products is considered; carbon in biomass added as inoculum and produced during fermentation is not considered.
